# Maxillary peripheral keratocystic odontogenic tumor. A clinical case report

**DOI:** 10.4317/jced.53438

**Published:** 2017-01-01

**Authors:** María del Carmen Vázquez-Romero, María de los Angeles Serrera-Figallo, Javier Alberdi-Navarro, Javier Cabezas-Talavero, Manuel-María Romero-Ruiz, Daniel Torres-Lagares, Jose-Manuel Aguirre-Urizar, Jose-Luis Gutiérrez-Pérez

**Affiliations:** 1 Master’s Degree in Oral Surgery - School of Dentistry - University of Seville; 2 Master’s Degree in Oral Pathology - Department of Stomatology II UFI11/25 School of Medicine and Dentistry - University of the Basque Country/EHU; 3 Private practice - Cáceres, Spain

## Abstract

The keratocystic odontogenic tumor is a benign odontogenic cystic neoplasia characterized by its thin, squamous epithelium with superficial parakeratosis. It has the potential for infiltration and local aggressiveness and has a high rate of recurrence. This neoplasia is predominantly found in males and people of white origin. The mandible is the most frequently involved site, in particular the third molar region, mandibular angle, and ramus. It has a mandible-maxilla ratio of 2:1. Only about twenty cases of peripheral keratocystic odontogenic tumors (PKCOT) have been reported in the international literature. This study presents a case of PKCOT localized in the anterior region of the maxilla, on the vestibular side of the upper left lateral incisor and the upper left canine. The diagnosis and treatment procedures, as based on the literature, are also discussed.

** Key words:**Odontogenic cysts, odontogenic tumors, keratocyst, keratocystic odontogenic tumor.

## Introduction

The keratocystic odontogenic tumor (KCOT) is a benign odontogenic cystic neoplasia characterized by its thin, squamous epithelium with superficial parakeratosis. It has the potential for infiltration and local aggressiveness and has a high rate of recurrence (1-3).

This neoplasia is predominantly found in males and people of white origin. It occurs mainly in the mandible, in particular the third molar region, mandibular angle, and ramus, with a mandible-maxilla ratio of 2:1.1 It can appear at any age; however, it is more frequent between the ages of 20 and 30. Its incidence rate ranges from 3 to 12% of odontogenic tumors (4). Similarly, this lesion can appear suddenly as a single clinical entity or as a complication of Gorlin-Goltz Syndrome (3).

Philipsen coined the term “odontogenic keratocystic” (OKC) for the first time in 1956 (4). The histopathologic criteria for diagno-sis of OKC were first established by Pindborg *et al.* (5) in 1962, in which particular attention was paid to its parakeratinazation. In 2005, the World Health Organization (WHO) reclassified the OKC as KCOT because of its clinical behavior and some genetic aspects (including local aggression, infiltrative growth, and a high rate of recurrence of up to 62.5%) (6).

Said tumor generally occurs intraosseously (1,7) and it is much less likely to grow extraosseously, in less than 0.5% of the cases described (6). Dayan *et al.* (8) described the term peripheral odontogenic keratocyst (POKC) in 1988, and it was later renamed as peripheral keratocystic odontogenic tumor (PKCOT) (4). According to the literature, to date only 22 cases of PKCOT have been reported, with these being primarily observed on gum tissue (17 out of 22 cases (8-18)), although it can also occur in the oral mucosa (only three cases have been described in the literature (19,20)) and in the lateral facial deep region (two cases reported (21)), with an occurrence rate of 77%, 14%, and 9%, respectively. In some cases, the lesion is characterized by an extraosseous side and a slight bone tissue invasion, a perforation of the cortical bone found under the PKCOT in some cases. Other cases are strictly extraosseous, with normal oral mucosa covering the PKCOT.

This study presents a case of PKCOT localized in the anterior region of the maxilla on the upper left lateral incisor and the upper left canine. The diagnosis and treatment procedures, as well as the main clinicopathological aspects, are also discussed.

## Case Report

A 32-year-old male presented a lump located in the anterior region of the left upper jaw. The patient had noticed a clear growth and self-reported three months of evolution. The patient’s primary concern was not only the increasing gum size itself, but also its esthetic ramifications, as the growth became visible upon smiling.

**Figure 1:**
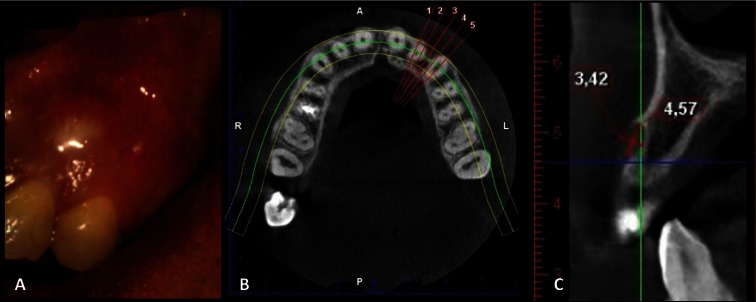
A) Intraoral view of the lesion B) CBCT: axial section and C) CBCT: sagittal section: lesion location, intraosseous and extraosseous involvement.

The intraoral examination revealed a whitish lump with a soft surface located between the upper left lateral incisor and the upper left canine (22 and 23). On palpation, the lump was fluctuant and not painful (Fig. 1). Teeth 22 and 23 were vital. A cone beam computed tomography (CBCT) was performed, revealing a well-defined, unilocular radiolucent lump of 4 mm in diameter, which was causing erosion of the vestibular cortical area.

Clinical and radiological aspects led to a presumptive diagnosis of non-inflammatory odontogenic cyst causing cortical perforation in the maxilla, which was also consistent with a gingival cyst of adult and keratocyst odontogenic tumor.

A cystectomy was performed under local anesthesia without conducting a root canal treatment of adjacent teeth, and the obtained material was sent for histopathologic testing.

A full-thickness incision was made, preserving the papilla to avoid future gum recession (papilla-base incision), beginning on the mesial part of 22 and ending on the distal part of 23. This incision continues with a vertical incision on the distal area of 23, passing the deformity, and penetrating the mucogingival junction to have better visibility and access. A full-thickness flap was carefully elevated to avoid tearing the flap or the cyst capsule. Once localized, the cyst was removed via curettage of the bone tissue.

After removal, the cyst was placed in 10% formalin and was sent to a pathological anatomy lab for histologic diagnosis. Subsequently, the flap was repositioned using a 6/0 non-absorbable suture.

The patient was prescribed antibiotic treatment (one tablet of amoxicillin/clavulanic acid 875mg/125mg every 8 hours for 7 days), as well as nonsteroidal anti-inflammatory drugs (600mg of ibuprofen; one tablet every 8 hours for 3 to 5 days). The patient was told not to brush in areas near the incision for the first 24 hours, and to use mouthwash with 0.12% chlorhexidine twice a day for two weeks. In addition, the patient was prescribed a soft, cold diet to avoid heavy chewing for at least two or three days. The stitches were removed two weeks later.

**Figure 2:**
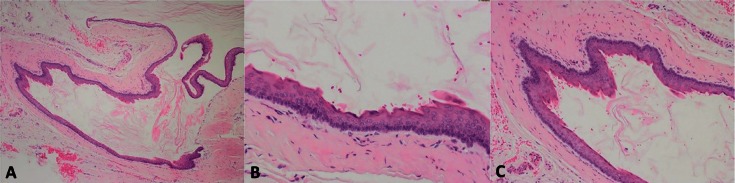
A) Cystic lesion, inner epithelial lining partially detached and cystic lumen contains keratin (H&E 4x). B) Simple squamous epithelium 4-8 layers with basal cells arranged in palisaded pattern and superficial parakeratosis, focally corrugated. Capsule formed by connective tissue (H&E 20x). C) Simple squamous cystic epithelium with palisaded basal cells and superficial corrugated parakeratosis (H&E 10x).

The histopathological findings revealed a cystic lesion with a loose connective tissue wall, an inner lining of well-defined simple squamous epithelium (4-8 cells in thickness), basal cells arranged in a palisaded pattern with a superficial parakeratinized focally corrugated surface. The epithelium was focally detached from the connective tissue, and keratin was found inside the cystic le-sion. The collected data established a peripheral keratocyst odontogenic tumor as the diagnosis (Fig. 2).

**Figure 3:**
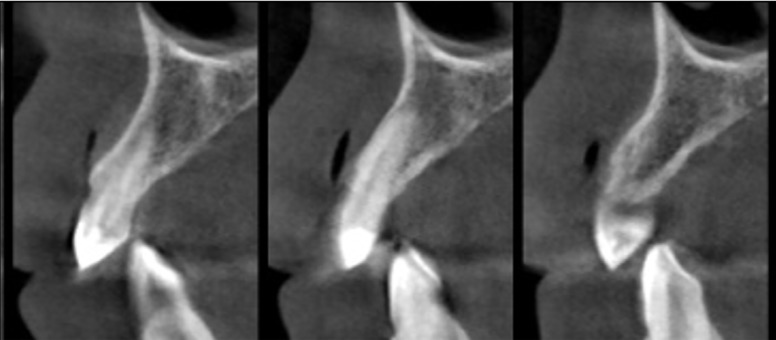
CBCT: Sagittal sections after a year: the surgical area shows signs of bone healing.

Periodic check-ups were carried out after one month, three months, six months and one year post-surgery, as well as a CBCT, which confirmed no signs of recurrence (Fig. 3). The patient was asked for their informed consent to publish their case, giving this consent.

## Discussion

Regarding the case’s clinical presentation, the cyst was surrounded by a smooth contour similar to a gingival cyst of adult, while the contour of the peripheral keratocyst odontogenic tumor (PKCOT) is usually irregular and undulating. The histological examination was successful, as there was evidence of “keratin scales.” In the majority of cases, this is very difficult to achieve. The lesion in this study revealed the histological characteristics of a PKCOT, including parakeratinization (8-21).

In the systematic review carried out in PubMed using the keywords “peripheral,” “keratocystic,” “odontogenic,” and “tumor,” only 22 cases of PKCOT were found, seventeen of which were localized on the gum tissue (77%), three on the oral mucosa (14%), and two in the lateral facial deep region (9%) ( Table 1).

**Table 1:**
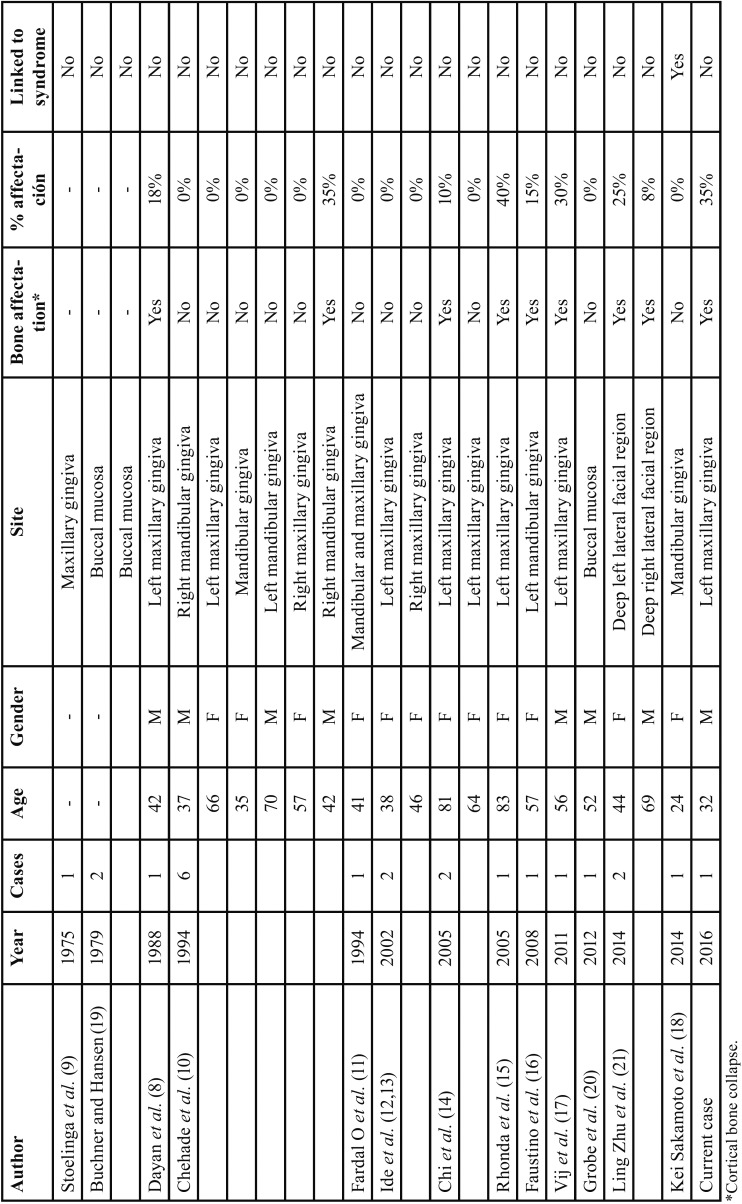
Summary of reported cases of peripheral keratocyst odontogenic tumor (PKCOT) in the literature.

Regarding the tumor’s location, the proportion of maxillary KCOT, with respect to those occurred in the mandible, is 1 to 2 or 1 to 3. The lesion is localized in the maxillary tuberosity in only 10% of cases, and with an even lower percentage in the canine area (21). PKCOT is most commonly found in the mandible, since there is a ratio of 12:7; there are 19 cases described in total, 12 of which occurred in the maxilla and 7 in the mandible ( Table 1). Regarding the vestibular or lingual location, it is more often found in the vestibular region, as was the case for the patient in this study (21). Likewise, it is important to highlight the esthetic consequences involved (22).

In addition, the tumor’s specific characteristics must be emphasized. Even though it was of relatively small size (approximately 4 mm), the tumor had perforated the vestibular cortical area. The size of PKCOTs can vary, but the most common dimensions are between 3 and 5 mm, and they are rarely larger, reaching even 3 to 4 cm (21). The link between these tumors and Gorlin-Goltz syndrome has been described, in which case the size is more commonly between 3 and 5 mm (18).

Controversy exists over whether PKCOT is a locally destructive lesion with a high rate of recurrence like KCOT, or whether it is an indolent lesion more similar to gingival cyst of adult (10-13). Ide *et al.* (12,13) affirmed that the PKCOT and the KCOT were not extraosseous and intraosseous variants within the same entity.

The question of whether or not the PKCOT is a counterpart of KCOT must be answered through exhaustive examination on a case-by-case basis. The PKCOT of the patient in this study seems to be a true expression of a KCOT in soft tissue (3,5,8).

In the cases linked to Gorlin-Goltz syndrome, PTCH1 mutations were identified, and the immunohistochemical results suggested that the KCOT and PKCOT lesions were caused by a genetic alteration in the patients’ PTCH1-GLI gene (18). As all cases of Gorlin-Goltz syndrome initially have mutations in PTCH, it is logical that these lesions appear.

Currently, there is no consensus on KCOT treatment due to its high rate of recurrence. As the literature shows, treatment of this tumor can range from marsupialization to a resection in-bloc, from most conservative to most aggressive treatments, respectively (2,7,10).

PKCOT cannot be completely compared to KCOT, as the clinical and biological behavior is not necessarily the same for both. Therefore, choice of treatment will depend on the age of the patient, the location and size of the tumor, and whether it is a primary or recurrent tumor. These factors will justify the differences found in the scientific literature (6).

In the present clinical case, a complete exeresis of the lesion was conducted with posterior curettage and a slight bone drill, thus avoiding the possibility of any remains of the lesions remaining in the affected area. To date, the patient presents no signs and symptoms of recurrence after a year.
